# Current Perspectives on Opisthorchiasis Control and Cholangiocarcinoma Detection in Southeast Asia

**DOI:** 10.3389/fmed.2018.00117

**Published:** 2018-04-30

**Authors:** Narong Khuntikeo, Attapol Titapun, Watcharin Loilome, Puangrat Yongvanit, Bandit Thinkhamrop, Nittaya Chamadol, Thidarat Boonmars, Teerachai Nethanomsak, Ross H. Andrews, Trevor N. Petney, Paiboon Sithithaworn

**Affiliations:** ^1^Cholangiocarcinoma Research Institute, Khon Kaen University, Khon Kaen, Thailand; ^2^Department of Surgery, Faculty of Medicine, Khon Kaen University, Khon Kaen, Thailand; ^3^Department of Biochemistry, Faculty of Medicine, Khon Kaen University, Khon Kaen, Thailand; ^4^Cholangiocarcinoma Foundation, Khon Kaen University, Khon Kaen, Thailand; ^5^Department of Epidemiology and Biostatistics, Faculty of Public Health, Khon Kaen University, Khon Kaen, Thailand; ^6^Department of Radiology, Faculty of Medicine, Khon Kaen University, Khon Kaen, Thailand; ^7^Department of Parasitology, Faculty of Medicine, Khon Kaen University, Khon Kaen, Thailand; ^8^Curriculum and Instruction Program, Faculty of Education, Khon Kaen University, Khon Kaen, Thailand; ^9^Faculty of Medicine, St Mary’s Campus, Imperial College, London, United Kingdom; ^10^Department of Ecology and Parasitology, Institute of Zoology, Karlsruhe Institute of Technology, Karlsruhe, Germany

**Keywords:** *Opisthorchis viverrini*, cholangiocarcinoma, screening, primary prevention, secondary prevention, tertiary care program

## Abstract

Similar to bile duct cancer or cholangiocarcinoma (CCA) in the western world, opisthorchiasis-associated CCA in Southeast Asia is an aggressive cancer with high mortality rates. It is known to cause a significant health burden in the opisthorchiasis region in Thailand and possibly throughout mainland Southeast. To reduce this health burden, a comprehensive prevention and control program for opisthorchiasis, as well as CCA, is required. In this review, our aim is to provide a brief update of the current situation regarding the natural history of opisthorchiasis and health burden of CCA in Southeast Asia. A comprehensive approach to tackling these issues being implemented in Thailand under the “Cholangiocarcinoma Screening and Care Program” is described. This comprehensive program consists of a three stage prevention and patient care program. The primary prevention component involves opisthorchiasis screening using a new and sensitive urine assay. The secondary prevention component involves screening for CCA and periductal fibrosis, with suspected CCA patients following the protocol for confirmation and appropriate treatment. Due to the eco-epidemiology of opisthorchiasis-induced CCA, the anticipated impacts and outcomes of the program include short-, medium-, and the long-term goals for the reduction of CCA incidence. To achieve long-term sustainable impacts, concerted efforts to raise social awareness and participating action by general public, non-government organizations, and government agencies are necessary. The strategic plans developed for this program can be expanded for use in other endemic areas as well as being a model for use in other chronic diseases.

## Introduction

The liver fluke, *Opisthorchis viverrini*, was recognized as a parasite of humans early last century; however, its role in causing cholangiocarcinoma (CCA) was officially recognized in 1994. It is one of three trematode species, the others being *Schistosoma haematobium* and *Clonorchis sinensis*, that are classified as group 1 carcinogenic parasites (1).

Our aim here is to provide a brief update of the current situation relating to opisthorchiasis and CCA in Southeast Asia and to discuss a comprehensive approach to tackling issues from primary and secondary to tertiary patient care based on a program that is being implemented in Thailand. Due to the eco-epidemiology of opisthorchiasis-induced CCA, the anticipated impacts and outcomes of the program include short-, medium-, and the long-term goals for the reduction of CCA incidence. To achieve such long-term, sustainable impacts, concerted efforts by general public, non-government organizations, and government agencies are required. This review consists of three main sections starting with a current update of opisthorchiasis and CCA, followed by intervention and solution, anticipated outcomes and long-term goals, and then conclusion and the future goals. As a case study, the comprehensive prevention and control program being conducted in Thailand will be illustrated. Finally the anticipated outcomes will be discussed in terms of the success, maintenance, and sustainability of the program. More details for specific issues regarding the natural history of opisthorchiasis and CCA can be found in some recent publications (2–5).

## Current Situation

### Distribution and Life Cycle of *O. viverrini*

**Figure 1:**
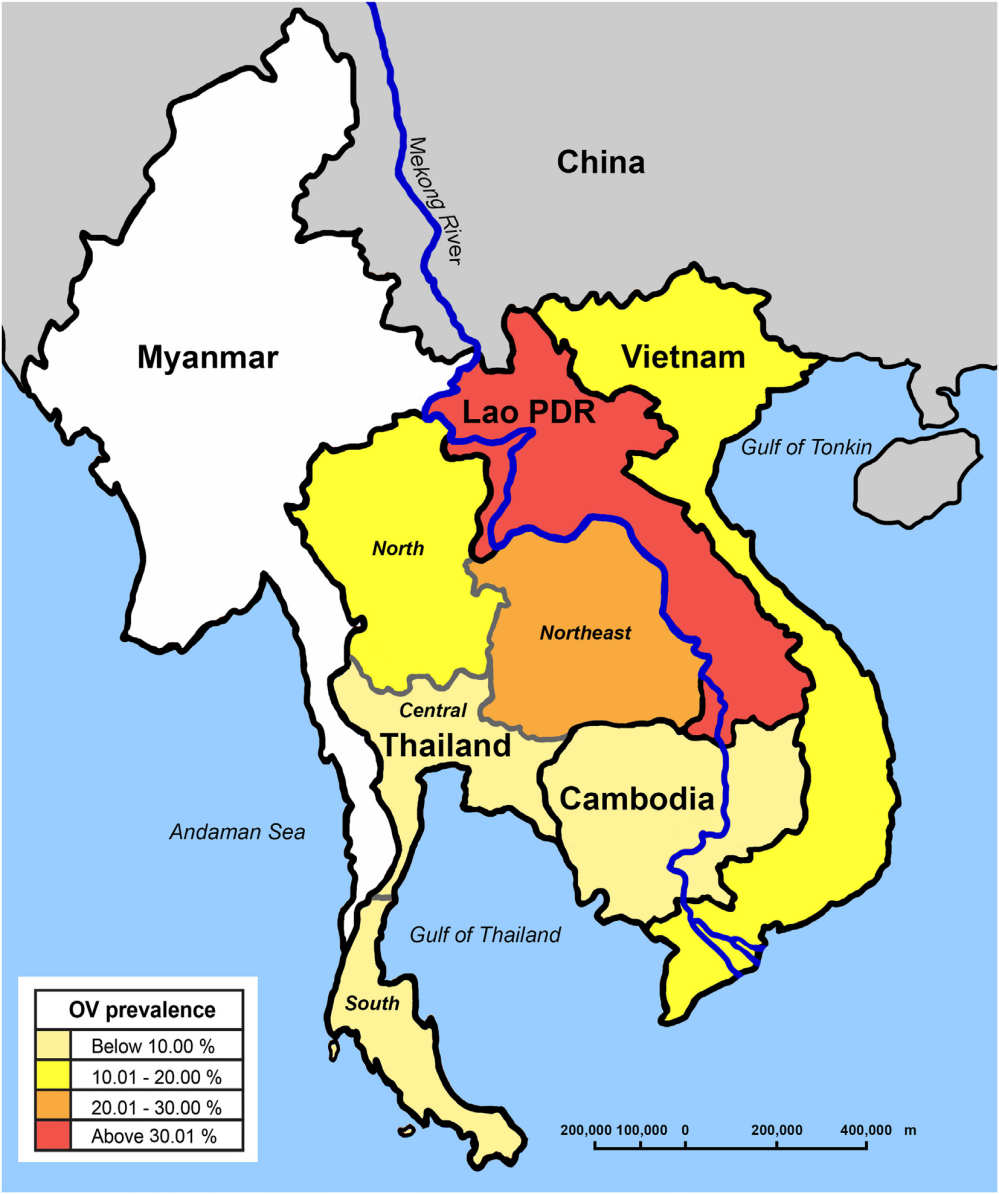
Distribution of *Opisthorchis viverrini* (OV) in Southeast Asia.

Substantial molecular and biological data exists showing that *O. viverrini* is a complex of morphologically similar species (6–8). *O. viverrini* sensu lato is found in those countries bordering the Lower Mekong River in continental Southeast Asia. It appears to be most frequent in Lao PDR, the north and northeast of Thailand, parts of Cambodia, and southern Vietnam (9), with a recent first record from Myanmar (10). Evidence suggests that its range is restricted to areas occupied by its first intermediate hosts, freshwater *Bithynia* snails (11) (Figure 1).

*Opisthorchis viverrini* has a complex life cycle in which eggs are excreted in the feces of the final hosts, usually humans, with cats and more rarely dogs also acting in this role (6, 12). The eggs are ingested, probably together with fecal matter, by freshwater snails that act as first intermediate hosts. To date, three snail taxa *Bithynia funiculata, Bithynia siamensis siamensis*, and *Bithynia s. goniomphalos* are known hosts, although molecular evidence suggests that *B. s. siamensis* and *B. s. goniomphalos* are full species within extensive species complexes (13). Eggs hatch in snails undergoing a phase of asexual reproduction before exiting as cercariae. They actively seek a cyprinid fish as second intermediate host developing to metacercariae that are infective to humans who eat uncooked or undercooked fish. Eating raw or mildly fermented fish is a traditional practice in the countries where *O. viverrini* is endemic (14). Once in the final host, the metacercariae develop to hermaphrodite adult worms and move into the biliary tract, attach to the epithelium, feed on host secretions, mate and lay eggs (15). Adult worms produce 50–200 eggs/g feces/day for many years (16).

### Molecular Biology and Systematics of *O. viverrini*

Molecular biology and systematics investigations of medically important trematodes, including the liver fluke *O. viverrini*, have used increasingly sophisticated methodologies that have increased our understanding of host–parasite interactions and the mechanisms of disease pathogenesis. Importantly, they provide a foundation to develop/establish new diagnostic methods, drug treatments, and effective vaccines. In addition, they enhance our understanding of the carcinogenesis process (17). Molecular studies have focused specifically on characterizing the *O. viverrini* protein tegument and excretion–secretion products, which can provide defined molecular targets to treat infection, facilitate the development of new, rapid/cheap diagnostic tools which could break the transmission cycle, and potentially identify targets for vaccine development (7).

Since the first report of liver fluke infection in prisoners in northern Thailand (18), only one species, *O. viverrini* has been recognized by morphology and geographical distribution (9, 15). A groundbreaking molecular genetic study by Saijuntha et al. (19) revolutionized *O. viverrini* systematics with the discovery that it is a species complex “*O. viverrini* sensu lato (sl)” (hereafter referred to as OV) containing two evolutionary lineages with many cryptic species (morphologically similar but genetically distinct) occurring in distinct wetlands in Thailand and Lao PDR. Furthermore, there is evidence of potential co-evolution between OV and its first intermediate host, *Bithynia* snails (13).

Since 2007, molecular genetic studies (RAPD, microsatellite, DNA, and RNA) have provided independent evidence for two genetically distinct evolutionary lineages existing in Thailand and Lao PDR, each of which contains many cryptic species with high genetic variability within and between different populations associated with different geographical areas, temporal factors, and fish host species (20–25). Apart from geographical factors associated with the genetic differentiation of OV populations, temporal and host interactions also have an effect on genetic variation, for instance, temporal factors and different fish species affect OV population genetics (21, 26). Furthermore, these studies showed that self-fertilization and/or a clonal distribution commonly occurs in populations/cryptic species. A recent molecular study (27) suggested that geographical separation is more important than fish host specificity in influencing OV genetic structure. Further studies should examine host selection for OV in *Bithynia* snails from different populations within each cryptic species, as they are the critical parasite amplifying stage. Furthermore, extensive molecular genetic studies are required to define the systematics and population structure of OV throughout continental Southeast Asia (6). An accurate understanding of the molecular biology and systematics of OV is essential for the prevention and control of infection and provides valuable information for early effective diagnosis and treatment of CCA, hence, reducing mortality rates in endemic areas (6, 8).

### Diagnosis and Screening of Opisthorchiasis

The conventional diagnostic method for human fish-borne trematodes, including OV, is searching for eggs in fecal samples. Several methods have been used successfully in the past, such as the modified formalin ether concentration technique (28), the modified thick Kato smear (29), and Stoll’s dilution egg count technique (30). The reliability of fecal examination was determined from an autopsy study that revealed many infected individuals with low infection intensities and limited egg output leading to an under diagnosis by as much as 20% (31). In addition to low infection intensities, intermittent egg excretion and bile duct obstruction due to chronic infection or cholangiocarcinogenesis all undermine the sensitivity of conventional stool examinations (32). Another diagnostic problem is the concurrent transmission of OV with several species of fish-borne zoonotic trematode belonging to the families Heterophyidae and Lecithodendriidae, referred to as minute intestinal flukes (MIF) (33–35). MIF eggs have a similar size and shape to the liver flukes eggs potentially causing false positive diagnoses, and reducing diagnostic specificity (15, 33). Molecular diagnosis of opisthorchiasis by PCR targeting at repeat DNA element offered high specificity but variable sensitivity (36–41). Loop-mediated isothermal amplification (LAMP) has been established for the detection of both OV and *C. sinensis* with a higher sensitivity than conventional PCR (42–44). Species-specific PCRs are now also available to distinguish between different liver fluke species: OV (40), *Opisthorchis felineus* (45) and *C. sinensis* (46). Molecular methods discussed earlier will contribute significantly toward a more effective and accurate diagnosis of trematode infections. Further simplification of the tests and an understanding of cost effectiveness under various socioeconomic scenarios are needed.

Alternatively, several serological antibody tests for opisthorchiasis and clonorchiasis have been developed as diagnostic assays with greater sensitivity and specificity than fecal examination. These include the intradermal test, immunoelectrophoresis, indirect hemagglutination assay, indirect fluorescent antibody test and indirect enzyme-linked immunosorbent assay (indirect ELISA) (39, 47). Indirect ELISA is preferred for the detection of antibodies using different types of antigen including crude somatic extracts of adult worms (48, 49) and excretory-secretory antigens (50, 51). These are superior to fecal examination. The detection of parasite-specific antibodies in other clinical samples, such as urine and saliva, is possible and offers the potential for the serodiagnosis of opisthorchiasis, and these antibodies could act as markers for associated morbidities (52–55). To increase diagnostic performance and reduce the cross reactivity of parasite proteins, several recombinant antigens from eggs and worms were produced and tested (56–59). However, our inability to discriminate current and past infections poses the main problem for serological antibody diagnosis (47).

To avoid the drawback of antibody-based detection, secretory products from adult worms could be used to indicate a current infection (60–62). In this regard, monoclonal antibody-based systems offer increased diagnostic sensitivity, as they discover infections when eggs are not detectable in fecal samples (61), as corroborated in an autopsy study (31). Currently, both copro and urine antigen detection are possible for opisthorchiasis, with the advantage of antigen detection when fecal examination for eggs is negative (63, 64). The antigen concentration measured is also correlated with the intensity of infection. Due to its simplicity and the non-invasive nature of sample collection, the urine antigen assay provides a better alternative diagnostic method to conventional fecal examination and has revolutionized the diagnostic approach of opisthorchiasis. The urine assay is being applied for large-scale population screening. It has become the method of choice in the control program in Thailand. Based on its diagnostic potential, a test strip in the form of the point of care test is now being developed and produced for large-scale use in endemic areas.

### Pathogenesis of Opisthorchiasis and Cholangiocarcinogenesis

Chronic infection and persistent inflammation are recognized as important risk factors in many human cancers; emerging evidence suggests that cancer-related inflammatory processes cause genetic instability and are involved in initiation, promotion, and progression of carcinogenesis (65). Opisthorchiasis, particularly repeated infections, causes chronic inflammation *via* pro-inflammatory cytokines (i.e., IL-6) and transcription factor NF-κB that induce oxidative stress response enzymes generating reactive oxygen and nitrogen species (ROS and RNS). The resulting persistent oxidative/nitrative stress disturbs the homeostasis of many adaptive response systems such as the oxidant/antioxidant ratio, DNA repair enzymes including many altered candidate genes involved in controlling cell proliferation, apoptosis, and fibrogenesis. Overproduction of ROS and RNS leads to genotoxic DNA damage as evidenced by the high level of 8-oxodG, 8-nitroguanine, and etheno-DNA adducts (εdA and εdC) detected in affected tissues. Excess ROS and RNS can also increase endogenous nitrosation reactions to yield carcinogenic *N*-nitrosamines such as NDMA that alkylate DNA bases. All these events may act as driving forces leading to cholangiocarcinogenesis. This has been intensively reviewed by Yongvanit et al. (66) and Thanan et al. (67). Some of these molecules (i.e., oxidized alpha 1-antitrypsin, 8-oxodG, etc.) are potential biomarkers for opisthorchiasis-associated periductal fibrosis and CCA (53, 68–71). However, reliable and specific biomarker(s) for opisthorchiasis-associated CCA remain to be determined.

### Health Burden, Morbidity, and Mortality

Cholangiocarcinoma is an uncommon cancer in most parts of the world, where incidences are usually lower than 2/100,000 cases/year (72). However, in areas where liver flukes are common parasites infecting humans, the incidence of CCA is much higher. Indeed, the highest worldwide are found in north and northeast Thailand, reaching between 98.8 and 317.6/100,000/year at ages above 35 years depending on the district examined (73). Accurate data from the other Southeast Asian countries where this parasite occurs are inadequate for determining the frequency of disease occurrence, although high prevalences of OV infection have been noted in parts of Lao PDR and Cambodia (74–77).

Infection with OV is mostly asymptomatic but can cause morbidity if the worm burden is high. In this case, symptoms may include right upper quadrant abdominal pain, flatulence, fatigue, and mild hepatomegaly (78). In very heavy infections, attached adult worms in the biliary tract can cause epithelial hyperplasia and potentially biliary obstruction leading to cholangitis, obstructive jaundice, periductal fibrosis, and enlargement of the gall bladder (79–81). If treated before CCA emergence, OV infection is usually of relatively minor medical significance and can be cured by use of the anthelmintic drug praziquantel.

**Figure 2:**
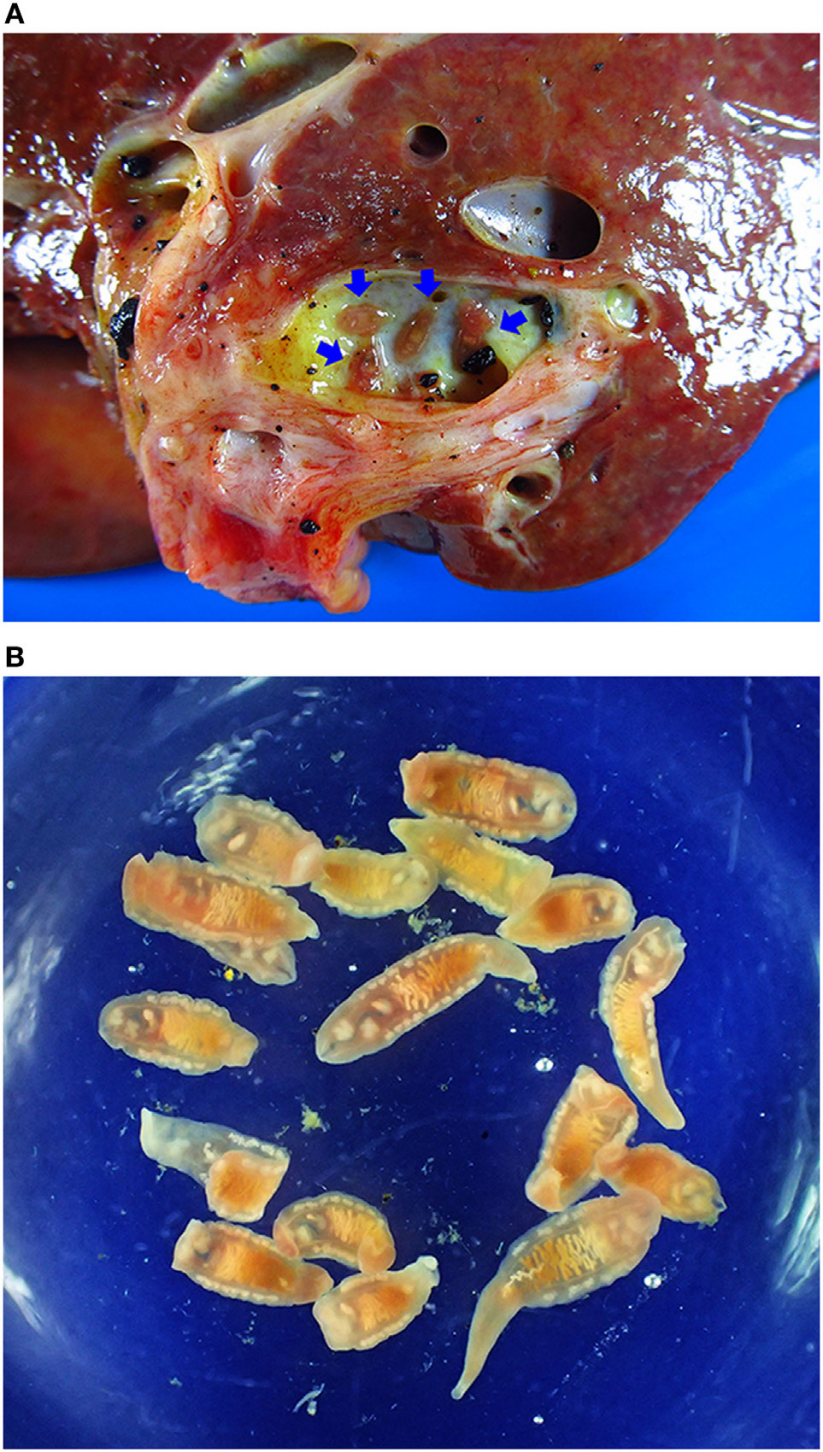
Opisthorchiasis-associated cholangiocarcinoma. **(A)** Cholangiocarcinoma specimen showing adult worms in the bile duct (blue arrows). **(B)** Adult *Opisthorchis viverrini* recovered from the liver.

**Figure 3:**
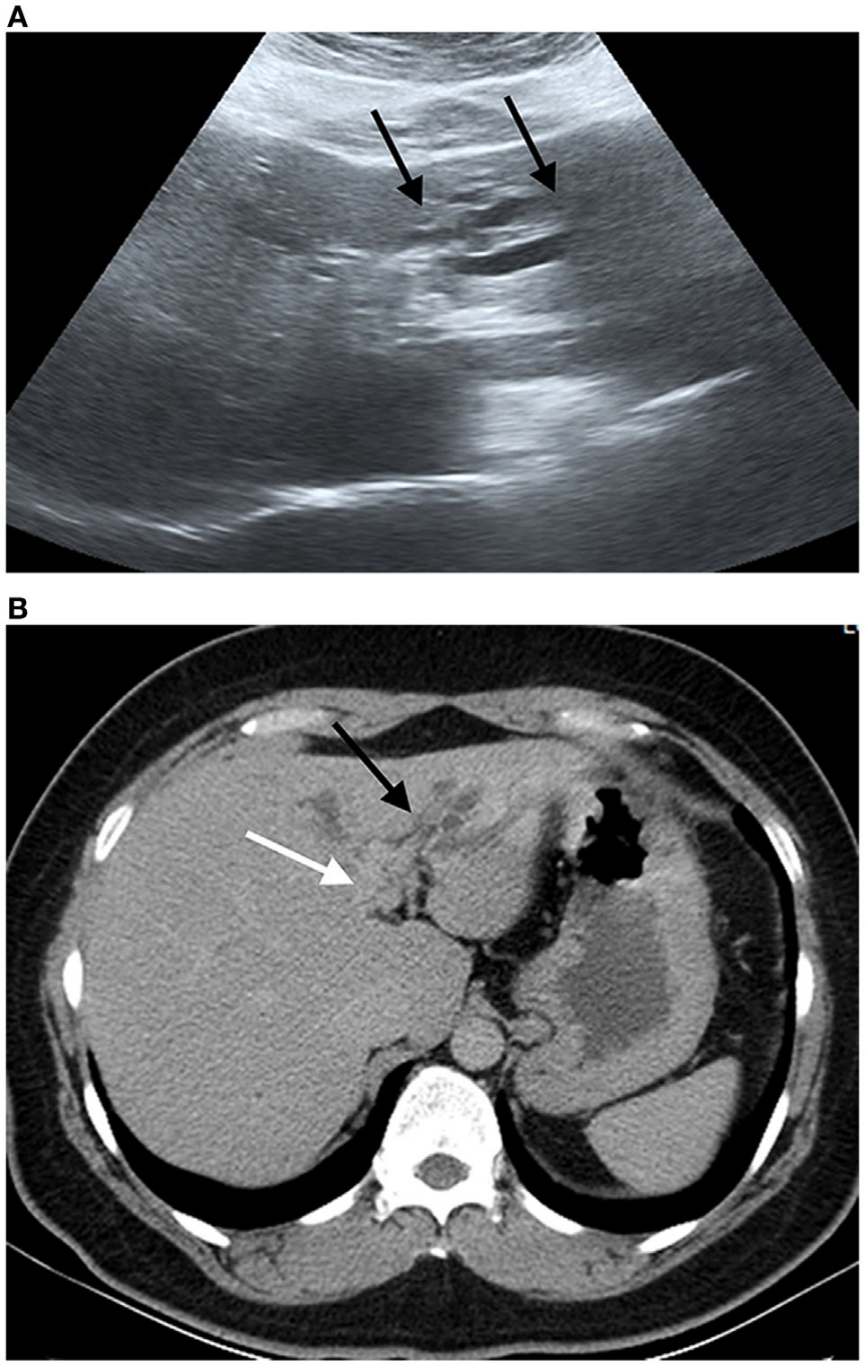
Ultrasound image and compute tomography in cholangiocarcinoma (CCA). **(A)** Ultrasound image shows a mild degree of dilatation of the intrahepatic bile duct left lobe of the liver (black arrows). **(B)** Portal phase computed tomography shows infiltrative tumor along left hepatic duct (white arrow) with dilatation of intrahepatic duct in left lobe (black arrow). Final diagnosis is perihilar periductal CCA stage II (AJCC 7th edition).

Early stage CCA is largely asymptomatic, so that patients presenting at a hospital usually have late stage disease with no curative option (82–84). CCA is by far the most common liver cancer in Thailand (85, 86), where it is the fifth most common cause of death in males and the eighth in females (87). Postmortem, a major burden falls on the family as death usually occurs in males in the 40–65 age group working in agriculture (88), i.e., individuals from relatively poor families who are breadwinners. An example of CCA patient who harbored live adult *O. viverrini* was illustrated in Figures 2 and 3.

## Intervention and Solution

### The Cholangiocarcinoma Screening and Care Program (CASCAP)

The CASCAP is a package with different levels of activities (Table 1). It consists of a primary control program targeting the prevention and control of opisthorchiasis. The ultrasound screening (US) for hepatobiliary disease including CCA serves as a secondary control program in which at risk population is systematically recruited for screening. Suspected patients are then sent for confirmatory diagnosis, treatment, and care in a tertiary control program.

**Table 1:** The missions of Cholangiocarcinoma Screening and Care Program and strategy to achieve specific goals.

Mission	Strategy/tool	Goal
Primary prevention	– Identify the current source of OV infection – New urine assay for diagnosis of opisthorchiasis – School-based curricula	– Block transmission – Effective screening and chemotherapeutic control of OV – Fluke-free generation

Secondary prevention	– Ultrasound screening of at risk individuals	– Early case detection and referral

Tertiary prevention	– Treatment – Palliative care	– Curative treatment and therapy

Isan Cohort	– Data center and management with ethical standards	– Public data base for disease control

Cholangiocarcinoma Foundation	– Raise funds to stimulate and support prevention and engage in public advocacy at all levels	– High social/public awareness of OV and CCA

### The Primary Prevention Program: Food Safety, School-Based Health Education, and Screening of *O. viverrini*

The primary prevention program involves a campaign for food safety education and an improved strategy for opisthorchiasis screening and treatment based on the urine assay. For the long-term vision, school-based curricula dealing with OV and CCA were initiated.

Because cyprinid fish are the source of infection, the primary preventive effort is to stop raw or insufficiently cooked fish being eaten. Food safety is the key issue for the control of opisthorchiasis, but this is difficult to reduce or stop the consumption if infective fish products as these have a long-standing tradition as key food items in the affected areas (14). Previous reports indicated that OV is still prevalent in different forms of fermented fish dishes in northeast Thailand (89) and “Pla som,” a short-term fermented cyprinid fish dish (3–4 days at ambient temperature) can contain viable metacercariae (89). This recent finding supports the hypothesis that Pla som is likely an important source of infection.

School-based health education has been advocated for prevention of many infectious diseases (90) as well as for OV and CCA (91). Based on our initial trial (92), a school-based curricula program was established through the Faculty of Education, Khon Kaen University (KKU), and included primary to secondary school courses as well as vocational study. This has been implemented in 31 schools in 3 provinces in northeast Thailand. Selected schools were located in active OV transmission in the Chi, Mun and Songkhram River wetlands, and the program spans 3 years with formal educational evaluation as well as the impact on OV infection.

The CASCAP screening program is being operated as a Thai Ministry of Public Health (MOPH) national program, from 2015 to 2026, as part of the national policy to control of opisthorchiasis and CCA. In 2015, the targeted population screened by fecal examination (Kato-thick smear) involved 76,000 people in 84 sub-districts in 27 provinces in the north, northeast, and central Thailand. The urine assay based from our previous work (64) is being applied in opisthorchiasis region nationwide to evaluate the current OV prevalence. It is anticipated that by using a more sensitive urine assay, the true prevalence of opisthorchiasis and its current distribution can be revealed. In addition, reinfection and new infections can be evaluated. Thus, the frequency of treatment can be planed for parasite control and eventually eliminate the parasite in the community.

### Secondary Prevention Program: US for CCA

Most patients presenting at a hospital have late stage disease with palliative care being the most common treatment option. Thus, there is a second, undetected group that has early stage CCA without being aware of it (88). If, however, these individuals with early stage disease can be found, then curative surgery is possible (93). To find these individuals, CASCAP was initiated (83, 88). Given that an estimated six million people in Thailand fall within threat risk group (age over 40, with a history of OV infection, eating raw or fermented fish, and/or relatives with CCA), the screening program is a massive undertaking.

Screening by US is a suitable method for the initial diagnosis of CCA, as well as for periductal fibrosis, which is a potential precursor of this cancer (94). The screening program started with the recruitment of at risk individuals who had previously registered at a sub-district health clinic. US examination takes place at local medical facilities (clinics and hospitals) or through the CASCAP mobile unit, which can examine 500 individuals a day. Screening divides the patients into four groups: (1) without liver pathology, (2) with liver pathology not directly related to CCA (e.g., fatty liver or cirrhosis), (3) with periductal fibrosis as a potential precursor of CCA, and (4) with suspected CCA (liver mass and bile duct dilatation). Patients with no pathology are asked to return for control in about a year, those with significant pathology not related to CCA are referred to a local hospital, those with periductal fibrosis are asked to return after 6 months for a control examination and those with suspected CCA are referred for further diagnostic tests.

A teleconsultation system, *via* the CASCAP Cloud software, is coupled with the US examination, allowing diagnostic confirmation by a specialized radiologist at a tertiary hospital (95). Digital images from the US examination are transferred to and stored on the Cloud system for future reference (88). The cohort studies will aid in the discovery of biomarkers for CCA and potentially speed up the process of screening.

### Tertiary Patient Care Program: Confirmation and Management of Suspected CCA

Ultrasound screening has had a major impact on active, early stage CCA detection and the subsequent treatment of patients, saving many lives. The initial results can be seen among the cohort that underwent screening, with a confirmed diagnosis of CCA. Among 54 patients with histopathologically confirmed CCA, the staging was 0–2 (72.5%) and 3–4 (27.5%). By contrast, in the walk-in cohort of 506 surgical cases (at Outpatient Departments), histopathological staging was 0–2 (29%) and 3–4 (71%). Although data confirmation is still required, this reverse trend from late to early stage CCA detected in the CASCAP program is a positive sign toward the eventual curative treatment and reduction of CCA.

The CCA Center of Excellence was established as a national, regional and international hub at Srinagarind Hospital, KKU. This contains 5 dedicated facilities: (1) a CCA ward (19 beds), (2) 1 operating theater, (3) an intensive care unit of 2 beds, (4) a bio-bank, and (5) research laboratories. This forms the basis for establishing five specialist CCA functions, namely, for tertiary referral, training, research, networking, and a national, regional, and international focal point. Currently, all five functions have reached their goals and are developing at a good level. Furthermore, a multidisciplinary CCA patient care team has been established to improve the quality of CCA care, which includes nurses, medical oncologists, hepatobiliary-pancreatic (HBP) surgeons, radiologists, pathologists, and radiotherapists. This provides avenues for a better understanding and effective dissemination of information, as well as for acquiring specialist skills at all levels to combat CCA.

To strengthen both screening and surgical capacity, capacity building and networking to cope with increasing number of CCA patients is needed. Two training programs were established to enhance screening and care. A Training Program in Cholangiocarcinoma US Screening for Radiologists and General Practitioners was initiated to provide radiologists and general practitioners with the knowledge, skills, and expertise to diagnose CCA using US. This training program operates every 2 months. More than 60 radiologists have passed the training program and can further train GPs and consult with GPs *via* teleconsultation (95). A total of 789 medical doctors from provincial and district hospitals throughout the country were trained to January 2018.

In Thailand, there are an estimated 20,000 CCA cases/year. Considering a single hospital, Srinagarind Hospital, unfortunately only about 10% of those affected (*ca*. 200 of 2,000) finally reached the hospital where they received appropriate treatment and palliative care (84). To increase the capability of the health-care system, particularly the provincial hospitals, improvement and expansion of facilities and personnel are inevitable. In particular, HBP surgeon training is a priority. Thus, a Training Program in Hepatobiliary and Pancreatic Surgery was initiated and is currently offered by the Department of Surgery, Faculty of Medicine, KKU. The objective is to provide trained surgeons with knowledge, skills, and expertise in the field of surgical diseases of the liver, biliary tract, and pancreas. This is a 1-year course, and up to eight HBP surgeons can be trained for work in north and northeast Thailand. The partner hospitals for surgical training included Khon Kaen, Sunpasitthiprasong, Surin Hospitals, and National Cancer Institute in Bangkok. In addition to the HBP training, special financial support from NGOs and charity organizations has been implemented to help increase the number of surgical treatments, i.e., 500 cases/year, within these network hospitals.

### Database Facility: Isan Cohort

The Isan Cohort is a web application located at www.cascap.in.th. This application uses the CASCAP protocol in an internet platform so that health-care institutes across Thailand can use it in a systematic, unified, single approach. The name “Isan” (the northeastern region of Thailand) is known to have the highest rate of CCA worldwide. The protocol started with the enrollment of at risk individuals. This created two initial cohorts: the OV Screening Cohort and the CCA Screening Cohort. Cohort members undergo the OV test followed by hepatobiliary US. Baseline demographic information and risk factors were recorded during enrollment, and screening results were recorded at US. If positive findings were detected, the OV Screening Cohort received praziquantel, while CCA Screening group patients were transferred for advanced diagnosis using CT/MRI. If confirmed, participants moved to the Patient Cohort. Information collected during these processes was recorded on case report forms designed by CASCAP.

In January 2018, there were 3,713 health-care centers voluntarily participated in the Isan Cohort study. Of these, 33 were tertiary hospitals, and 354 were community hospitals equating to 44% (387/881) of all hospitals in Thailand. The remaining 3,326 were health-care centers. Each center applied for an account at Isan Cohort where the Isan Cohort generated a new database and assigned to a Uniform Resource Locator (URL) specifically for each center. The URL took the name of the institute in place of the www part of the Isan Cohort URL. Hence, Isan Cohort become the central “parent” site with an unlimited number of “daughter” sites so that privacy within each institute remained preserved even though health-care information exchange (HIE) was possible.

Second, the institutes can transfer health information in their own health information system (HIS) to Isan Cohort automatically in real time. This can be done by installing the Thai Database Connector (TDC) in their replicated HIS servers. The TDC triggers algorithms that can transfer required data that are encrypted in the Isan Cohort server. This is done automatically once the data are entered into HIS. Thus, work is not replicated, allowing HIE without borders. In addition, this approach ensures sustainability of the Isan Cohort even if there is limited financial support because stored health information can be updated without any additional effort as long as the institutes remain members of Isan Cohort. To date, 23,618,285 individuals have the health information required by CASCAP archived at Isan Cohort. Of these, the member institutes enrolled have 1,406,216 individuals. Baseline information on demographics and risk factors are available for 771,659 individuals. The US screening has been done on 421,895 individuals. Of these, 38.5% had a diagnosed liver abnormality (Table 2). Approximately two-fifths were diagnosed with fatty liver disease and periductal fibrosis (19.7 and 17.4%, respectively). Only 0.5% was diagnosed with cirrhosis and 1.4% with liver parenchymal change. A total of 4,358 (1.0%) individuals were diagnosed with suspected CCA and referred for further investigation.

**Table 2:** Number and percentage of ultrasonography diagnoses (*n* = 421,895).

Ultrasonography findings	No.	%
Normal liver	259,465	61.5
Abnormal liver	162,429	38.5
Fatty liver (combined grading)	83,102	19.7
Periductal fibrosis	73,303	17.4
Cirrhosis	2,279	0.5
Parenchymal change of liver	5,809	1.4
Liver mass	4,601	1.1
Bile duct dilated	4,999	1.2
Suspected CCA	4,358	1.0
Gall stone	16,303	3.9
Renal cyst	14,639	3.5
Renal stone	11,906	2.8

## Anticipated Outcomes and Long-Term Goals

On 26th December 2014, as opposed to the top down policy in the past, the agenda for opisthorchiasis and CCA control was initiated by the Cholangiocarcinoma Foundation of Thailand and the National Health Security Region 8, with strong support by citizen groups, and passed to the National Health assembly where the motion was approved and endorsed by the Cabinet of Ministers of Thailand on 8th May 2015.

### Policy Implementation

On 21st June 2016, the Thai MOPH announced a decade long policy to eliminate OV and reduce CCA. The vision is for Thai citizens to be safe from OV and CCA and to have a better quality of life. It encourages preventive behavior, and the risk groups for CCA are provided with adequate and holistic medical care until cure or final stage disease. A set of strategic plans was laid down to cover activity related to: (1) intensive surveillance of OV and CCA, (2) strengthening preventive strategies in Thailand and neighboring countries, (3) enhancing screening, care, and referral systems, (4) supporting and facilitating community and local authority participation in the prevention and management of OV and CCA, and (5) strengthening research and development for efficient comprehensive data base management. As a result of these plans, the cumulative targets over the next 10 years for OV screening and treatment are >4 million, US screening >5 million, and up to 15,000 cases for surgical treatment. Based on the number of CCA victims/year, the cumulative numbers for CCA surgery and treatment are clearly less than the annual number of CCA cases. However, the initiative is expected to create an environment stimulating a more concerted effort and willingness of all parties concerned to move forward in a desirable direction.

### Social Participation and Mobilization: Cholangiocarcinoma Advocacy *via* the Cholangiocarcinoma Foundation of Thailand

Since 1950 government-driven programs have been carried out across Thailand to combat liver fluke infection based on parasitic disease control and cure. However, these have had only limited success in reducing the incidence of OV infection, and subsequently CCA. Moreover, some disadvantaged patients have no access to healthcare. Therefore, the Cholangiocarcinoma Foundation of Thailand was established on the 28th of September 2012 with the aim of mobilizing the public sector directly in the fight to prevent and cure CCA and to save millions of lives.

Khon Kaen University is centrally located in the endemic area with the highest incidence of CCA worldwide. The Faculty of Medicine provides the underlying foundation, personnel, and infrastructure for the Cholangiocarcinoma Research Institute, the CASCAP, the CCA Center of Excellence, which provides diagnostic and clinical services to CCA patients, as well as the Cholangiocarcinoma Foundation of Thailand. These interdisciplinary centers help to facilitate research preclinically and clinically and serve as specialty care centers for patients at risk of or suffering from CCA.

### Model and Area Expansion

Health problems consist of multiple causes and are multidimensional. The CCA screening program is also active in diagnosing diabetes, hypertension, kidney and additional liver diseases, etc. Thus, the CACAP model, with its strong population data based software, can also serve as a basis for non-communicable disease control, for example, the diagnosis of chronic kidney diseases, in northeast Thailand. A similar extension can be made for diabetes, hypertension, and heart disease.

The current global community is involved in population movement and migration, including borderless economic communities such as ASEAN, leading to transborder disease movement. The success of OV and CCA control within a single country, Thailand, can never be ensured without the close collaboration of the neighboring countries where OV is endemic: Lao PDR, Cambodia, and Vietnam. The CASCAP model can and should be extended to these countries.

## Conclusion and Future Goals

Carcinogenesis leading to CCA development takes a considerable time (potentially many years and likely decades) after liver fluke infection. Many advances have been made in the past 20 years toward understanding parasite population biology, epidemiology, and control, as well as pathogenesis and eventual cholangiocarcinogenesis. The prevalence and intensity of OV infection has shown some reduction due to control activities and improved sanitary conditions, but CCA incidence remains more or less unchanged. This may simply reflect a lack of priority. With the massive mortality that it causes, a comprehensive and multidisciplinary control approach is required. The CASCAP model has been successfully used to tackle primary, secondary, and tertiary care with a huge data base deriving from the Isan cohort. There has also been a substantial role played by non-government/charity organizations. In particular, since OV control and reduction of CCA incidence takes time, the challenge is to reduce the risk of CCA in the threatened population. The best way is to stop exposure to OV infection and the creation of a fluke-free generation. Indeed, it is likely that until the young, fluke-free generation has replaced the current middle-aged generation, CCA will continue to be a problem. In addition to government policy and support, social awareness is fundamental for control momentum and sustainability, such that OV and CCA are perceived as everybody’s problem.

Because of the uniqueness of the CCA etiology in Southeast Asia, which differs markedly from that of Western countries, new and specific research ventures are required to battle the disease. An urgent and badly needed research outcome is to facilitate the screening of the risk population to OV, as well as to CCA. While robust biomarkers for OV screening, i.e., the urine assay, have made initial inroads, similar biomarkers for CCA are being discovered. An additional research dimension is to expand and modify the control program into nearby countries where OV is endemic, such that comprehensive and concerted efforts can be made toward the programs long-term goals, and a future fluke-free generation will be a reality.

## Author Contributions

All authors contributed to writing and reviewed the entire manuscript. Different sections were predominantly written by authors as follows; Sections “Distribution and Life Cycle of *O. viverrini*” and “Health Burden, Morbidity, and Mortality” (TP); Section “Molecular Biology and Systematics of *O. viverrini*” (RA); Section “Diagnosis and Screening of Opisthorchiasis” (PS); Section “Pathogenesis of Opisthorchiasis and Cholangiocarcinogenesis” (PY and WL); Section “The Primary Prevention Program: Food Safety, School-Based Health Education and Screening of *O. viverrini*” (PS, TB, and TN); Section “Secondary Prevention Program: US for CCA” (NC); Section “Tertiary Patient Care Program: Confirmation and Management of Suspected CCA” (NK, AT, and NC); Section “Database Facility: Isan Cohort” (BT); Section “Policy Implementation” (NK and PS); Section “Social Participation and Mobilization: Cholangiocarcinoma Advocacy *via* the Cholangiocarcinoma Foundation of Thailand” (PY); and Sections “Introduction,” “Model and Area Expansion,” and “Conclusion and Future Goals” (NK, TP, and PS).

## Conflict of Interest Statement

The authors declare that the research was conducted in the absence of any commercial or financial relationships that could be construed as a potential conflict of interest.
